# The master role of polarized NIS expression in regulating iodine metabolism in the human body

**DOI:** 10.20945/2359-3997000000583

**Published:** 2023-03-13

**Authors:** Bernadett Lévay, András Lantos, István Sinkovics, András Slezák, Erika Tóth, Orsolya Dohán

**Affiliations:** 1 National Institute of Oncology Multidisciplinary Head and Neck Cancer Center Budapest Hungary National Institute of Oncology, Multidisciplinary Head and Neck Cancer Center, Budapest, Hungary; 2 National Korányi Institute of Pulmonology Department of Pathology Budapest Hungary National Korányi Institute of Pulmonology, Department of Pathology, Budapest, Hungary; 3 National Institute of Oncology Department of Nuclear Medicine Budapest Hungary National Institute of Oncology, Department of Nuclear Medicine, Budapest, Hungary; 4 National Institute of Oncology Department of Molecular Pathology and Surgical Pathology Center Budapest Hungary National Institute of Oncology, Department of Molecular Pathology and Surgical Pathology Center, Budapest, Hungary; 5 Semmelweis University Department of Internal Medicine and Clinical Oncology Budapest Hungary Semmelweis University, Department of Internal Medicine and Clinical Oncology, Budapest, Hungary

**Keywords:** NIS, thyroid gland, iodine absorption, iodine metabolism

## Abstract

**Objective:**

The aim of this study was to investigate how polarized sodium iodide symporter (NIS) expression may regulate iodide metabolism *in vivo*.

**Materials and methods:**

Polarized NIS expression was analyzed in tissues that accumulate iodide by the use of immunohistochemistry and polyclonal antibody against the C-terminal end of human NIS (hNIS).

**Results:**

Iodide absorption in the human intestine occurs via NIS expressed in the apical membrane. Iodide is secreted into the lumen of the stomach and salivary glands via NIS expressed in the basolateral membrane and then circulates back from the small intestine to the bloodstream via NIS expressed in the apical membrane.

**Conclusion:**

Polarized NIS expression in the human body regulates intestinal-bloodstream recirculation of iodide, perhaps prolonging the availability of iodide in the bloodstream. This leads to more efficient iodide trapping by the thyroid gland. Understanding the regulation and manipulating gastrointestinal iodide recirculation could increase radioiodine availability during theranostic NIS applications.

## INTRODUCTION

Iodine is an essential component of thyroid hormones which, in turn, are key factors for human physiology and development before and after birth. Active iodide transport into thyroid epithelial cells is mediated by the sodium iodide symporter (NIS), a plasma membrane glycoprotein. Diagnosis and therapy of thyroid diseases using radioiodine have been applied since 1940 (1,2), but NIS was only cloned in 1996, in the laboratory of Nancy Carrasco (3-5).

A secondary structure model for NIS, with 13 transmembrane segments, has been proposed based on experimental data (6-8). NIS couples the inward translocation of sodium and the simultaneous inward “uphill” translocation of iodide against their electrochemical gradients. The sodium gradient that provides the driving force for cellular iodide uptake is maintained by the sodium-potassium ATPase. Two sodium cations are transported for each iodide anion (9). In the thyroid, both NIS and sodium- potassium ATPase are located in the basolateral surface of thyroid follicular cells, close to the gland's blood supply (8,10,11).

Thyroid-stimulating hormone (TSH) and iodide are the two main factors regulating iodide transport in the thyroid, which is stimulated by TSH and decreased by iodide. Hence, TSH stimulation and iodide depletion are the two most important modulators routinely used to optimize radioiodine treatment in metastatic thyroid carcinoma (8,12,13).

NIS-mediated radioiodine therapy for thyroid cancer is the oldest routinely applied molecular targeted radiotherapy available today. Currently, the *NIS* gene is one of the most promising candidates for gene therapy applications, both as a therapeutic and a reporter gene (8,14).

A major drawback of available traditional cytotoxic anticancer therapies is their lack of selectiveness for cancer cells and their substantial toxicity against normal cells. Therefore, the ultimate aim of any new anticancer therapy is to achieve selective destruction of cancer tissue with minimal harm to healthy cells. One of the most promising approaches to accomplishing this is targeted radiation therapy. Radioiodine therapy is a prime example of targeted radiation therapy via selectively expressed plasma membrane transporters (2,14,15). Radioiodine therapy has been employed with great success for over 60 years for the destruction of thyroid cancer remnants and metastases after thyroid surgery. The presence of NIS in thyroid cancer cells ensures that the administered radioiodine accumulates selectively in these cells, thus causing little damage to other cells and only minimal side effects. Thus far, radioiodine therapy has been viewed as applicable only to thyroid cancer. However, NIS can concentrate various radionuclides in target cells and facilitate exciting applications, including diagnostics and gene therapy (11,14). Indeed, recent observations have raised the possibility of the application of radioiodine therapy to breast cancer and other cancers by the introduction of *NIS* into tumors via viral vectors or upregulating the tumors’ endogenous *NIS* expression, if present. As a transgene, *NIS* can be used for image-guided radiotherapy, monitoring of gene and vector biodistribution, and evaluation of trafficking of therapeutic cells. A potential limitation of ectopic (extrathyroidal) NIS expression is the fact that extrathyroidal tissues are unable to perform iodide organification. Therefore, the accumulation of iodide is the sum of cellular uptake and efflux, largely dependent on the plasma availability (absorption and clearance) of the tracer (8,11,14,16).

NIS is a master molecule of iodine metabolism. In rodents, NIS has been shown to be responsible for intestinal iodide absorption (8,17). Some of the absorbed iodide undergoes organification in the thyroid. Iodide is secreted via NIS in the salivary glands, stomach, and gastrointestinal lumen and is then again absorbed via NIS in the small intestine. Iodide is finally excreted by the kidneys via glomerular filtration (5,8).

Polarized NIS expression in epithelial cells results in vertical transepithelial transport of iodide. Polarization of NIS expression may occur across all cells, as NIS is located in the basolateral plasma membrane in all tissues where it is expressed, except for enterocytes in the small intestine, where NIS is confined to the apical membrane (17,18). NIS localized in the basolateral membrane transports iodide into the cell lumen, while NIS localized in the apical membrane transports iodide from the lumen into the cells. Interestingly, NIS is regulated differently in each of these tissues. Currently, apical expression of NIS in the small intestine epithelium has only been demonstrated in rodents (17).

Based on these considerations, the aim of our study was to follow the distribution of radioiodine in the human body by using whole-body single-photon emission computed tomography (SPECT) imaging and correlate the findings with NIS immunohistochemistry in human tissues that accumulate radioiodine. We were particularly interested in evaluating NIS expression in the human small intestinal epithelium and understanding the possible role of NIS in iodide absorption and recirculation.

## MATERIALS AND METHODS

NIS can be considered a master molecule of iodine metabolism. We studied the polarized NIS expression in tissues that accumulate iodide by using immunohistochemistry and a polyclonal antibody against the C-terminal end of NIS. Notably, NIS is expressed in the basolateral membrane of epithelial cells from salivary glands, thyroid, and stomach, while in the intestinal epithelium, the expression of NIS occurs in the apical membrane. NIS is regulated differently in each of these tissues and is responsible for iodide absorption from the intestine. Some of the absorbed iodide undergoes organification in the thyroid. In the gastrointestinal lumen, iodine is secreted via NIS into the stomach and salivary glands and is again absorbed via NIS in the small intestine. The role of this recirculating mechanism of iodine in the gastrointestinal system remains unclear. Iodine is finally excreted by the kidney via glomerular filtration.

In the present study, we performed immunohisto-chemistry of NIS using paraffin-embedded samples.

This study was approved in 2020 by the local ethics committee of the National Institute of Oncology in Hungary.

### Imaging

**Figure 1:**
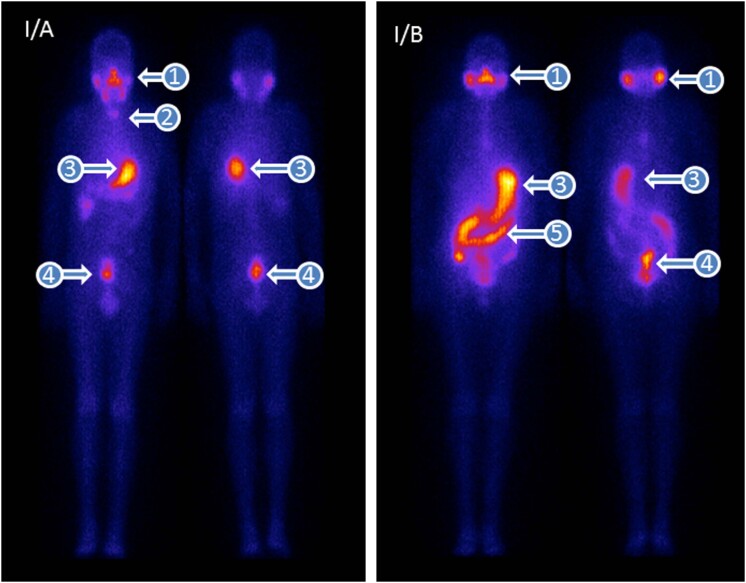
Planar images of post-therapeutic radioiodine-131 distribution in a patient who had undergone thyroidectomy due to thyroid cancer. The images were obtained 72 hours after the therapeutic administration of radioiodine-131. (1) Salivary gland, iodide is transported into the saliva; (2) thyroid remnant, iodide is trapped in the thyroid gland; (3) stomach, iodide is transported into the gastric juice; (4) iodide, as an anion, undergoes glomerular filtration into the urine in the bladder; (5) from the stomach, the gastric juice progress to the small intestine, where iodide is reabsorbed from the intestinal lumen and transported to the bloodstream.

Radioiodine distribution in the human body was evaluated using planar whole-body imaging after therapeutic administration of radioiodine-131 in patients who had undergone thyroidectomy for cancer. Post-therapeutic whole-body scan is routinely performed in anterior and posterior images (Figure 1).

A whole-body scanner was used to image the distribution of radioiodine-131 across the entire body. In the case of radioiodine-131, a high-energy collimator (up to 450 keV) was used with a scanning speed of 12 cm/min and a window setting of 364 keV ± 15%. Counts were obtained from the regions of interest over the entire body and thyroid in anterior and posterior images. Geometric averages for anterior and posterior counts were used. The counts were calibrated to the counts at time *t* < 60 min before any voiding.

### NIS immunohistochemistry

The local Institutional Review Board of the National Institute of Oncology reviewed and approved the immunohistochemistry examination of stored paraffin- embedded tissues. Human NIS (hNIS) polarized expression was studied in iodide accumulating tissues by immunohistochemistry using a polyclonal antibody against the C-terminal end of hNIS (6,11), as previously described (16,19,20). Briefly, 5-µm sections were sliced from stored surgical blocks of iodide accumulating tissues obtained from the thyroid, salivary gland, stomach, and small intestine. All slides were deparaffinated, rehydrated, and subjected to antigen retrieval. Endogenous peroxidase activity and biotin activity were blocked using a commercial blocking system (Ventana Medical Systems, Tucson, AZ, USA). Sections were stained using anti-hNIS rabbit polyclonal antibody (1 µg/mL in 1:4000 dilution) directed against the C-terminal end (a generous gift from Dr. Nancy Carrasco) using a commercial immunohistochemistry kit (Ventana Medical Systems). All slides were counterstained with hematoxylin.

## RESULTS

**Figure 2:**
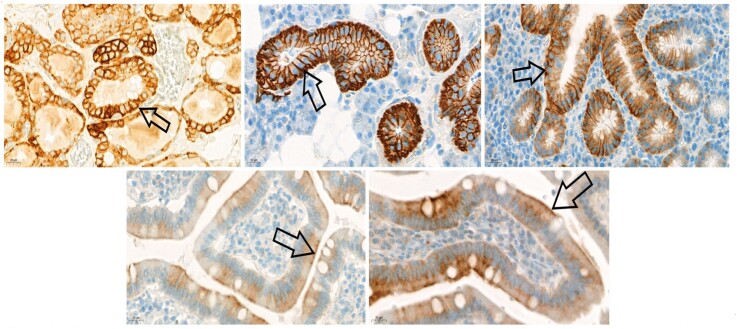
Sodium iodide transporter (NIS) immunohistochemistry. Black arrows show basolateral membrane staining indicating iodide accumulated in the follicular epithelium in the thyroid gland (top left); basolateral membrane staining indicating iodine excreted by epithelial cells in salivary gland acini (top middle) and in the gastric mucosa (top right). Apical membrane staining of the epithelial lining on the surface of duodenal villi responsible for iodide absorption (bottom left: cross-sectional, bottom right: longitudinal plane; NIS immunohistochemistry, 40x magnification).

Routine planar imaging during whole-body radioiodine distribution showed an accumulation of iodide (via NIS) in the stomach, salivary glands, and gastrointestinal lumen. Iodine dissolved in the gastric juice progresses to the duodenal lumen and is absorbed via NIS in the small intestine, in a similar process described in rodents. No significant radioiodine accumulation was observed in the distal part of the small intestine or in the colon. In the circulation, iodide is trapped by the thyroid (when this gland is present) or finally excreted by the kidney through glomerular filtration (Figure 2).

The results of NIS immunohistochemistry showed the localization of the NIS protein in the basolateral membrane in epithelial cells from salivary glands, thyroid, and stomach and in the apical membrane in epithelial cells from the small intestine (Figure 2).

The images showing the distribution of radioiodine correlated well with NIS polarized expression. The NIS expressed in the apical membrane was responsible for iodide absorption from the intestine. In the stomach and salivary glands, iodide was secreted into the lumen by NIS expressed in the basolateral membrane; then, in the small intestine, iodide circulated back to the bloodstream via apical NIS (Figure 2).

## DISCUSSION

We have correlated the *in vivo* distribution of radioiodine in the human body and the polarized NIS expression using immunohistochemistry in iodide accumulating organs. Iodine is a trace mineral in the environment and is ingested by humans via food. Because of its scarcity, iodine must be absorbed through a very specific and efficient mechanism. Iodide anions are quickly filtered from the bloodstream by the kidney and excreted via urine. Iodide is also “trapped” by the thyroid; it is transported by NIS into thyrocytes, where it is covalently bound to the tyrosyl residues of thyroglobulin. NIS is highly specific and efficient in accumulating iodide into thyroid cells, but iodide is also quickly cleared from circulation by glomerular filtration. Thus, it is critical that iodide remains in circulation for a sufficiently long time (17,21).

Nicola and cols. were the first authors to demonstrate NIS expression across the entire length of the small intestine in rodents (22). The authors analyzed the segment from the duodenum to the ileum and found that NIS is located in the apical surface of small intestine enterocytes where it mediates intestinal iodide absorption. They also reported that – similar to NIS expressed in the thyroid – enteral NIS is autoregulated by iodide and that high iodide concentration decreases the intestinal transport of this ion (17,23).

In this study, we used immunohistochemistry to show apical NIS expression in enterocytes for the first time in the human small intestine. The findings of our study corroborate the previous work by Nicola and cols., which was performed in rodents (22).

Studies of human samples are limited by availability and tissue autodigestion, and thus we could not evaluate *NIS* expression in the small intestine. Our samples, obtained from the duodenum, showed apical NIS expression in the enterocytes. It is difficult to study the regulation of expression and polarized plasma membrane targeting enteric NIS expression in humans. Martín and cols. reported the presence of a highly conserved monoleucine-based sorting signal in the NIS carboxy-terminus, which is responsible for basolateral plasma membrane targeting in polarized canine kidney epithelial (MDCK) cells. Disrupting this basolateral sorting signal results in apical targeting of NIS protein in epithelial cells. This determinant must be recognized by sorting machinery that is cell-specific and characterized by distinct adaptor proteins.

**Figure 3:**
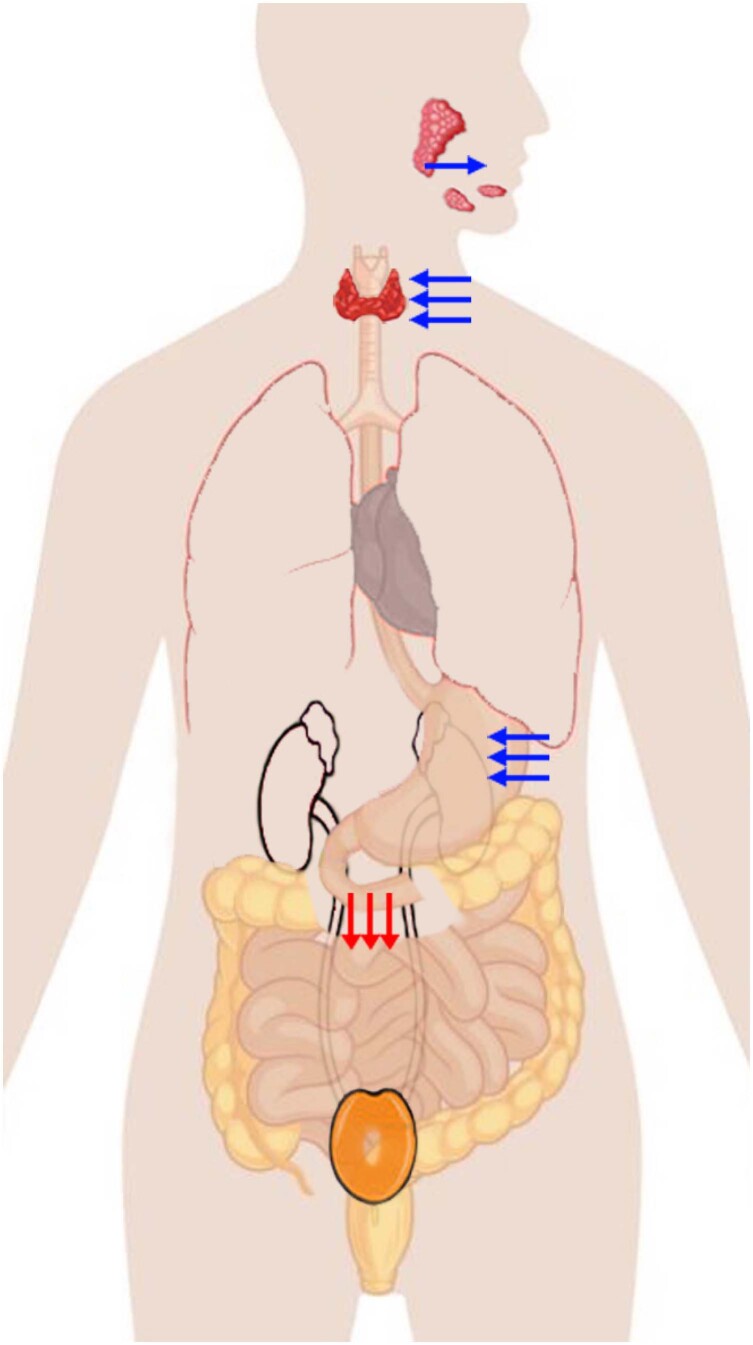
Schematic representation of iodine metabolism. Iodide distribution in the human body is regulated by tissue-specific polarized (basolateral or apical) plasma membrane expression of human sodium iodide transporter (NIS) in epithelial cells. Blue arrows: NIS expressed in the basolateral membrane in epithelial cells transporting iodide into the saliva, gastric juice, and thyroid cells, where it is covalently bound to the tyrosyl residues of thyroglobulin. Red arrows: NIS expressed in the apical membrane in the epithelial cells of the duodenal mucosa transporting iodide from the intestinal lumen to the bloodstream.

Very little is known currently about NIS-expressing tissues regarding the presence, function, and regulation of cell-specific factors that interact with and determine the polarized targeting of NIS in the plasma membrane (18,24). In the human body, polarized NIS expression may occur in the basolateral membrane (thyroid, salivary gland, stomach) or apical membrane (small intestine). This distribution ensures that the iodide absorbed in the small intestine accumulates in the thyroid and is secreted into the gastric lumen. Iodide is then reabsorbed in the small intestine to be finally removed from circulation to the urine through glomerular filtration (Figure 3).

In conclusion, iodine and TSH are the two main factors regulating thyroidal iodide transport, in which the transport is stimulated by TSH and decreased by iodine (3-5). Hence, TSH stimulation and iodine depletion are the two most important modulators routinely used to optimize radioiodine treatment in metastatic thyroid carcinoma.

Both tissue-specific expression and polarized NIS expression determine the distribution of iodide in the human body. As an anion, absorbed iodide is quickly filtered from the circulation by the kidney into the urine. Gastrointestinal recirculation can lead to prolonged plasma retention of iodide, thus resulting in higher iodide availability and accumulation into the thyroid. NIS is the first theranostic molecule ever used, as it is used in thyroid imaging, as a reporter gene in gene therapy, and in radioiodine treatment of metastatic thyroid cancer. Manipulating intestinal recirculation of iodide can optimize radioiodine availability in the bloodstream for NIS-mediated targeted radiotherapy and reporter imaging.
